# Rapid, Multiplexed Characterization of Shiga Toxin-Producing *Escherichia coli* (STEC) Isolates Using Suspension Array Technology

**DOI:** 10.3389/fmicb.2016.00439

**Published:** 2016-05-20

**Authors:** John M. Carter, Andrew Lin, Laurie Clotilde, Matthew Lesho

**Affiliations:** ^1^Pacific West Area – Western Regional Research Center – Produce Safety and Microbiology Research, Agricultural Research Service, United States Department of Agriculture, Albany, CAUSA; ^2^ORA/PA-FO/SAN-LB – Office of Global Regulatory Operations and Policy – Oceans, Reefs & Aquariums – Food and Drug Administration, United States Department of Health and Human Services, Alameda, CAUSA; ^3^MagArray, Inc., Milpitas, CAUSA; ^4^Luminex Corporation, Austin, TXUSA

**Keywords:** *E. coli*, Shiga toxin, immunoassay, PCR, microbead

## Abstract

Molecular methods have emerged as the most reliable techniques to detect and characterize pathogenic *Escherichia coli*. These molecular techniques include conventional single analyte and multiplex PCR, PCR followed by microarray detection, pulsed-field gel electrophoresis (PFGE), and whole genome sequencing. The choice of methods used depends upon the specific needs of the particular study. One versatile method involves detecting serogroup-specific markers by hybridization or binding to encoded microbeads in a suspension array. This molecular serotyping method has been developed and adopted for investigating *E. coli* outbreaks. The major advantages of this technique are the ability to simultaneously serotype *E. coli* and detect the presence of virulence and pathogenicity markers. Here, we describe the development of a family of multiplex molecular serotyping methods for Shiga toxin-producing *E. coli*, compare their performance to traditional serotyping methods, and discuss the cost-benefit balance of these methods in the context of various food safety objectives.

## Introduction/Background

In the U.S., Shiga toxin-producing *Escherichia coli* (STEC) represent a significant public health concern causing approximately 175,000 illnesses annually (Scallan et al., 2011). The CDC estimates the annual U.S. costs for acute care of STEC patients to be $1–2 billion (Paton and Paton, 2000 Science and Medicine p 28–37). Furthermore, STEC-related recalls are expensive for the food industry. For example, the outbreak of *E. coli* O157 associated with spinach from California’s Salinas Valley in 2006 cost farmers up to $200 million (Arnade et al., 2010). Although *E. coli* O157 remains the most common STEC serogroup in the U.S. (Centers for Disease Control and Prevention [CDC], 2011), over 200 additional *E. coli* serogroups exist (Hussein and Bollinger, 2005). Those non-O157 STEC are responsible for over 60% of the STEC infections (Scallan et al., 2011). The clinical manifestations of STEC infections range from mild watery diarrhea to severe complications of hemorrhagic colitis (HC), hemolytic uremic syndrome (HUS), and even death (Levine et al., 1987; Gyles, 2007). Since not all STEC appear to cause illness, distinguishing pathogenic STEC from the ones that do not pose a health risk remains a current challenge for regulatory agencies worldwide. Certain STEC serogroups are more highly correlated with severe illness than others. For example, STEC O26, O45, O103, O111, O121, and O145 are referred to as the “Big Six" and account for 75–80% of non-O157 STEC isolations in clinical samples in the U.S. (Gould et al., 2013). In addition, STEC O91, O113, and O128 have also been previously reported to cause HC and HUS (Johnson et al., 2006), and an STEC O104-caused outbreak in Germany in 2011 sickened 3816 individuals, making it one of the largest HUS outbreaks ever reported (Muniesa et al., 2012). Thus rapidly identifying STEC belonging to those serogroups is as important for protecting consumer health as early diagnosis of STEC infection for determining the proper treatment (Gould et al., 2009). Additionally, such rapid identification can alert public health officials that an outbreak has occurred and aid in matching clinical, food, and environmental isolates when attempting to trace back to the source of contamination.

Traditional serotyping employs O-specific antisera, usually in a slide agglutination format. These methods are quite simple, robust, and rapid. However, they can be both labor-intensive and time-consuming for large numbers of isolates, and may lead to ambiguous results because of variability in antisera production and a lack of standard methodology. Whereas slide agglutination is also relatively inexpensive when only a few of the most common antigens are tested, maintaining a complete set of hundreds of antisera reagents for O serotyping is expensive and most often left to central reference laboratories adding to the time of analysis (Dijkshoorn et al., 2001). Currently, there is a need for faster tests. One solution is to allow a food sample to be probed simultaneously for specific STEC serogroups, which can then be extracted from that food sample, thus completing detection and isolation of STEC in foods within 24 h. Suspension array technology using microbeads has been useful in filling this need by enabling development of rapid, high-throughput adaptable assays for identifying clinically relevant STEC O-serogroups. These microbead-based assays can theoretically analyze up to 500 targets in a single reaction, so targets can be added or removed as needed. For example, a 7-plex immunoassay was developed by Clotilde et al. (2013) to identify O serogroups O26, O45, O103, O111, O121, O145, and O157. The microbeads were also capable of binding to live cells, which aides in isolating pathogenic organisms for further characterization studies (Clotilde et al., 2013). Another example is a 13-plex PCR-based suspension assay to identify 11 O-serogroups: O26, O45, O91, O103, O104, O111, O113, O121, O128, O145, and O157; and two virulence factors: *eae* and *aggR* (Feng et al., 2015). These two assays are fast (<4 h), high throughput (96 well format), and were able to identify STEC serogroups more accurately than traditional serotyping (Clotilde et al., 2015).

## Suspension Array Technology

Microbead-based suspension array technology has emerged as a standard method for simultaneously detecting multiple biological analytes from one sample and has enabled a wide variety of applications in life science research, clinical diagnostics, food safety, and biodefense (McBride et al., 2003; Toro et al., 2013; Sun et al., 2014; Di Cristanziano et al., 2015; Khalifian et al., 2015; Silbereisen et al., 2015; Zhang et al., 2015; Kong et al., 2016). Commercial assay kits are available for cytokine profiling, infectious disease diagnostics, genotyping for inherited diseases, food pathogen typing, organ transplant compatibility testing, and many more. Many of these kits are built on the Luminex^®^ xMAP^®^ multiplexing platform, which utilizes fluorescent dyes to create sets of microbeads with unique spectral identities. Unique capture molecules are coupled to each set of microbeads, which then capture different analytes of interest. After binding to a detector molecule, which subsequently binds to a fluorescent reporter, these microbeads are read in a flow cytometer such as the Luminex^®^ 200^TM^ or FLEXMAP 3D^®^ instrument, or in a bead imager such as the Luminex MAGPIX^®^ instrument to determine both the presence and quantity of analyte(s) in the sample. The Luminex systems are capable of detecting up to 50, 100, or 500 different analytes from one sample in the MAGPIX, Luminex 200, or FLEXMAP 3D instruments, respectively. Some advantages of suspension array platforms include fast kinetics due to mobile capture surfaces, broad dynamic range (>3 logs), and high precision due to multiple independent measurements on many microbeads for each analyte in the sample. Open platform suspension array systems also enable researchers and other biomedical professionals to design and build tests for their specific applications. In the area of food safety discussed here, an important aspect of suspension array technology is the ability to detect DNA, RNA, and protein targets with the same system.

## Immunoassay/Protein Based Serotyping

Inoculation of *E. coli* produces a strong immune response, which targets immunodominant antigens, such as lipopolysaccaride (O-antigens) and flagellum (H-antigens). The best-known *E. coli* serotype is O157:H7. Strains sharing the O157 O-antigen (i.e., members of the O157 serogroup) also tend to share pathogenic phenotypes. For example, most strains belonging to the O157 serogroup exhibit prophages that code for Shiga toxin (Stx). Other serotypes may also carry Stx-encoding phage, and in the US seven STEC serogroups are considered adulterants in foods: O26, O111, O103, O121, O45, O145, and O157. Although there are particular strains within these serogroups that do not produce Stx, all are grounds for regulatory actions, including product recalls. Thus the food industries have considerable interest in *E. coli* serogroup testing.

**FIGURE 1:**
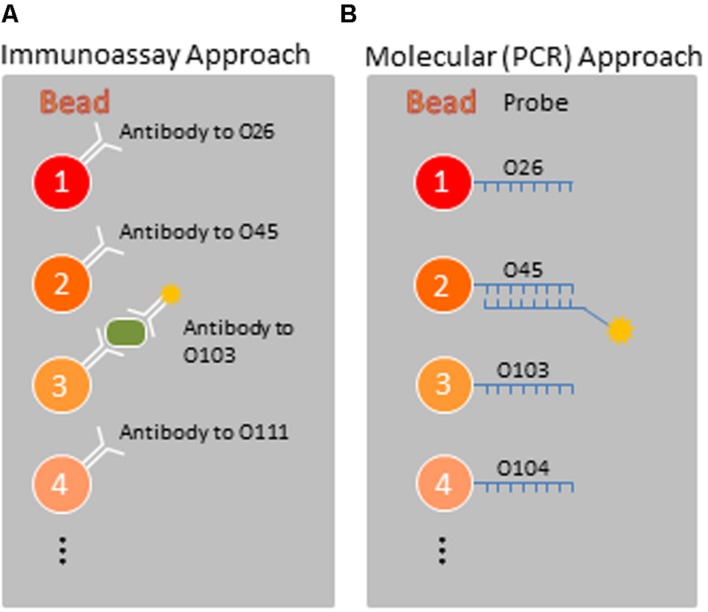
**Two microbead-based approaches for characterization of STEC. (A)** Sandwich immunoassay techniques targeting cell-surface antigens. **(B)** Molecular (gene-based) techniques targeting specific genetic markers of serogroup and virulence. Only four microbead regions are shown, with each region targeting a specific STEC serogroup.

Identification of specific *E. coli* serotypes is important for tracing infections to their environmental source. Outbreaks were formerly detected and defined solely by the serogroup of pathogens, and serotyping still provides a rapid and inexpensive means for preliminary characterization. Until recently the “gold standard” for serotyping was slide agglutination with O and H antigen specific antisera (Orskov et al., 1977; Machado et al., 2000). Now, alternative immunochemical assay formats are also available, including our Luminex-format microbead suspension array as a solid phase for multiplex typing of various STEC serogroups. (See **Figure 1A**).

Our assay architecture is similar to a sandwich ELISA, starting with an antibody (Ab) that is bound to a solid phase support. In a typical ELISA, this “capture Ab” is non-covalently adsorbed to a microplate well, but we use covalent attachment. When the target is added, the capture antibody can bind and pull its antigen out of the solution phase. Next the detector Ab is added, which binds to the antigen to create the Ab sandwich configuration. Finally the detector Ab is detected by means of a probe. In ELISA, that probe is labeled with an enzyme. In our assay, the probe is labeled with phycoerythrin, a bright fluorophore. Because bacteria exhibit many copies of O-antigen, the same Ab may be used for both capture and detection.

To generate the assay reagents, magnetic microbeads were covalently coupled to capture Ab according to the instructions provided with the BioRad Amine Coupling kit (BioRad, Hercules, CA, USA), using an amount of Ab based on a preliminary microplate ELISA data (1–10 μg/mL). This reaction is a common two-step carbodiimide protocol with N-hydroxysulfosuccinimide. Coupling was confirmed using phycoerythrin-labeled anti-species Ab. Detector Ab were biotinylated with the EZ-Link Sulfo-NHS-Long Chain-Biotin kit (Pierce, Rockford, IL, USA), according to the manufacturer’s instructions. Coupled microbeads and detector Ab were stable at 2–8°C for up to 1 year.

Samples were generated by blending foods at a 1:10 dilution into Brain Heart Infusion broth. A 1mL aliquot was then spiked with ~10 CFU of *E. coli* and incubated overnight shaking at 25 or 37°C. Appropriate BSL2 precautions were used when handling live pathogens. For example, samples were spiked after blending, and cultures were handled in screw-cap tubes, to reduce aerosolization.

For the assay, incubations were all 1 h at room temperature, in black microplates, swirling at 100 rpm. Washes were all threefold, using phosphate-buffered saline (PBS), pH 7.4, containing 0.05% Tween 20 (PBS-T) and a Bio-Plex Pro Wash Station (BioRad). For each sample, a 100 μL aliquot was combined with 5000 of each type of microbead, then incubated and washed. The microbeads were then resuspended in 100 μL of a cocktail containing 4 μg/mL of each biotinylated detector Ab, then incubated and washed. The microbeads were then resuspended in 100 μL of 4 μg/mL streptavidin labeled with R-phycoerythrin (SAPE), incubated and washed. The microbeads were finally resuspended in 100 μL PBS and then analyzed on Luminex 100 or MAGPIX instruments.

For these experiments, we used overnight cultures, and we were not concerned with assay sensitivity. We typically recovered 50–90% of microbeads from samples, depending on the matrix. We found that even 100 microbeads (instead of 5000) were sufficient for robust and reproducible signals. In validation experiments performed with three strains of each of the seven listed STEC serogroups, the assay gave 100% sensitivity and specificity (Clotilde et al., 2013). When we expanded the validation to include 161 environmental STEC strains our Luminex immunoassay missed only one strain of O157. In this latter experiment we compared performance of our assay to standard assays and a microbead-based PCR serotyping assay (Clotilde et al., 2015). This comparative study (**Table 1**) suggested that our Luminex immunoassay also missed 10 strains of O111 and O128. However, these targets were not included in our 7-plex immunoassay.

**Table 1:** List of disagreements between serotyping methods.

		Luminex		Additional serotyping	
Strain #	Conventional	PCR	Antibody	Statens	Remel
4	OUT	O157	O157		O157
5	OUT	O157	O157		O157
8	OUT	O157	O157		O157
11	OUT	O157	O157		O157
60	OUT	O157	O157		O157
83	OUT	O157	O157		O157
116	OUT	O157	O157		O157
126	OUT	O157	O157		O157
120	OUT	O45	O45		
25	OUT	O111	–	O111	
32	OUT	O111	–	O111	
69	O127	O128	–	O128	
71	O125	O128	–	O128	
72	O111	O128	–	O128	
73	O125	O128	–	O128	
74	O127	O128	–	O128	
75	O127	O128	–	O128	
77	O127	O128	–	O128	
70	O166	O128 and O121	O121	O128 and O121	
86	O125	O157	O157		O157
105	O158	O157	O157		O157
88	O137	O111	O111	O111	
85	O157	O157	–		

*OUT, other untypeable.*

We have now further expanded our assay to include the 10 most common STEC in the US: O26, O45, O103, O111, O121, O145, and O157, plus O91, O113, and O128. We still observe excellent sensitivity and specificity. We believe that STEC will continue to evolve additional pathogenic serogroups in the future. Regulatory agencies currently base their actions on the serogroup of such emerging adulterants, but there is consensus toward surveillance of virulence factors, rather than O-antigens. We already have working immunoassays for Stx (Clotilde et al., 2011), and we plan to add intimin, a virulence factor involved in *E. coli* attachment.

## Molecular Serotyping

The O antigen, a polymer of repeating oligosaccharides, is a component of the lipopolysaccharide of gram negative bacteria, and is used for serotyping *E. coli* into O serogroups. The *wzx* flippase, and *wzy* polymerase genes code for proteins involved in making the O antigen oligosaccharide, have proven to be O serogroup specific, and thus excellent targets for O serogroup specific PCR assays (Lin et al., 2011b). These genes were used to develop a multiplex suspension array that can identify 11 STEC O serogroups: O26, O45, O91, O103, O104, O111, O113, O121, O128, O145, and O157 (Lin et al., 2013) (**Figure 1B**).

Primer and probe sequences from each of the 11 O serogroup *wzx* or *wzy* genes were identified. Primers were designed with one biotinylated primer, and one unlabeled primer to result in a biotinylated amplicon. A single primer mix with primers for all targets was prepared. PCR reactions were carried out according to Lin et al. (2013). Signal to noise was improved with asymmetric PCR amplification with concentrations of biotinylated: unlabeled primer at a ratio of 5:1. Probe sequences complimentary to the biotinylated strand were conjugated to Luminex MagPlex^®^ microbeads according to the manufacturer’s instructions (Luminex Corporation, 2007). After multiplex PCR, amplicons were hybridized to labeled microbeads and incubated with SAPE. Reactions were then analyzed with a Luminex compatible cytometry-type instrument to interrogate each uniquely colored microbead and detect the amount of reporter molecule. Median fluorescent intensities (MFI’s) were calculated for each analyte, and a signal to background ratio was determined, where background is the MFI measured using one or more wells containing all ingredients except for template DNA. Signal to background ratio was calculated using Bio-Plex Manager software as (Sample MFI-Background MFI)/Background MFI. Samples are considered positive when signal to background ratio is greater than 5.0.

The PCR based Luminex suspension array for O serotyping has proven to be accurate, robust, and adaptable. A panel of 114 STEC isolates were all correctly identified, while 46 non-STEC and non- *E. coli* yielded no false positives (Lin et al., 2011a). In a multi-laboratory study of blind samples involving nine laboratories, a total of 492 isolates were identified correctly out of 495 analyzed (99.4% accuracy) (Lin et al., 2013). Another advantage of the suspension array system and of PCR based suspension arrays is the flexibility to add or remove targets from the array as needed. For instance, the 11-plex suspension array to identify 11 STEC O serogroups was recently modified to include STEC attachment factors that may be important markers of pathogenic potential. Primers and probes for the *eae* intimin gene, and the *aggR* regulator of the enteroaggregative phenotype have been added to the 11-plex O serotyping array to comprise a 13-plex STEC suspension array (Feng et al., 2015).

Suspension array technology allows for rapid, accurate, high throughput analysis of STEC isolates. In addition to identifying the most clinically relevant STEC O serogroups, the 13-plex suspension array is able to detect the presence of adherence factors that are associated with HUS, allowing regulatory and public health labs to determine pathogenic potential. The addition of the virulence factors to the suspension array also illustrates the flexibility of suspension array technology. While the big six STEC O serogroups and the O91, O128, O113, O104 additional O serogroups are presently the most concerning, new emerging O serogroups could be added to the suspension array. Additional targets could be added to the array, if other genes prove to be more highly correlated with illness. For instance, the H flagellar antigen is a useful phylogenetic marker (Ju et al., 2012) and could be added to the array to indicate the full O and H serotype. Other putative virulence factors that could be included in an STEC virulence profile include the plasmid-encoded enterohemolysin (*ehxA*), STEC autoagglutinating adhesin (*Saa*), extracellular serine protease (*espP*), long polar fimbria (*lpf*), and a bifunctional catalase-peroxidase (*katP*) (Law, 2000; Etcheverria and Padola, 2013). Another area of future study is to evaluate the effectiveness of the suspension array in screening food, environmental, and clinical samples. A preliminary study of artificially inoculated fresh produce and raw milk resulted in over 90% agreement between the suspension array and qPCR screening for *stx1* and/or *stx2* genes (Kase et al., 2015). Further improvements such as including an internal amplification control would be useful especially when screening enrichments to ensure that PCR inhibition does not cause false negative results.

## Discussion

Both of these suspension array assays represent improvements in speed and accuracy for detection and characterization of STEC pathogens in foods. A unique feature of the microbead-based immunoassay is that the magnetic microbeads pull down live pathogens, concentrating them. We have found that positive samples are easily cultured from the material remaining in the assay microplate (Clotilde et al., 2011). This facilitates further characterization, e.g., via molecular experiments. Other possibilities include use of an orthogonal detection scheme, other than SAPE. One group has combined immunomagnetic pull-down on Luminex microbeads with a modified fluorescence in situ hybridization (FISH) detection method (Stroot et al., 2012). This capture antibody-targeted FISH assay (CAT-FISH) method provides sensitivity equal to the Luminex immunoassay with the possibility of additional specificity from the nucleic acid-based FISH reporter. Among the many advantages of these suspension arrays is the ability to quickly upgrade and enhance assays as new genomic information becomes available for other pathotypes. Beyond the addition of more informative targets, other potential enhancements for these assays include workflow simplification, improved reagent stability through lyophilization or stable, engineered antibodies, and the ability to interrogate single cells with the assay, which would enable the characterization of mixed populations of STEC.

These assays fit on the spectrum of STEC characterization techniques between single-plex PCR assays for rapid *E. coli* screening of food samples and Next-Generation Sequencing (NGS) techniques. NGS is benefitting assay developers by providing information about novel molecular targets to more completely characterize STEC samples. While NGS is a powerful tool in microbiological research and outbreak investigations, there is still a need for low-complexity multiplex assays to make specific determinations about prioritized serogroups. One of the most significant drawbacks to fluorescent microbead-based methods is the cost of reagents, specifically the microbeads themselves. If we test 100 samples with a 10-plex assay, then 1000 assays are performed, and the cost per assay is acceptable. However, if half of those samples are identified as O157, then a more cost-effective work flow might begin with slide agglutination for all samples, using a single-plex O157 assay, followed by work-up of the non-O157 samples using a more expensive multiplex assay. The tradeoff of this workflow is that the time taken to do a full determination of the non-O157 samples is increased significantly. The suspension array technology still remains a versatile platform that benefits research, industrial, and regulatory labs by enabling a variety of protein and genetic analyses for many foodborne pathogens of interest (e.g., sets of assays to identify and characterize *Salmonella*, STEC, and foodborne toxins).

## Author Contributions

JC Drafted and edited immunoassay/protein based serotyping section and discussion. He and his lab staff conceived, developed, built, and tested the assays that resulted in the data in **Table 1**. AL Drafted and edited the Introduction and Molecular Serotyping sections. He conceived and developed the molecular serotyping assays described herein, including the enhanced assay with markers of pathogenic potential. LC Provided significant editorial contribution to all sections. Collaborated with JC and AL in building and testing the assays described. ML Outlined the article, drafted abstract and suspension array sections and formulated key messages in the discussion section. Luminex Corporation currently manufactures *E. coli* Serogroup Identification assays.

## Disclaimer

This presentation is not intended to be or appear as an endorsement, either directly, or indirectly, by the U.S. Government of any product or service described in this presentation. The U.S. Government does not directly or indirectly endorse any product or service presented or described in this presentation. The U.S. Department of Agriculture (USDA) prohibits discrimination in all its programs and activities on the basis of race, color, national origin, age, disability, and where applicable, sex, marital status, familial status, parental status, religion, sexual orientation, genetic information, political beliefs, reprisal, or because all or part of an individual’s income is derived from any public assistance program. (Not all prohibited bases apply to all programs.) Persons with disabilities who require alternative means for communication of program information (Braille, large print, audiotape, etc.) should contact USDA’s TARGET Center at (202) 720-2600 (voice and TDD). To file a complaint of discrimination, write to USDA, Director, Office of Civil Rights, 1400 Independence Avenue, SW, Washington, DC 20250-9410 or call (800) 795-3272 (voice) or (202) 720-6382 (TDD). USDA funding was administrated through the Agricultural Research Service, National Program 108 Food Safety

## Conflict of Interest Statement

The authors declare that the research was conducted in the absence of any commercial or financial relationships that could be construed as a potential conflict of interest.
